# Protein Kinase C as a Therapeutic Target in Non-Small Cell Lung Cancer

**DOI:** 10.3390/ijms22115527

**Published:** 2021-05-24

**Authors:** Mohammad Mojtaba Sadeghi, Mohamed F. Salama, Yusuf A. Hannun

**Affiliations:** 1 Department of Biochemistry, Molecular and Cellular Biology, Stony Brook University, Stony Brook, NY 11794, USA; mohammad.sadeghi@stonybrook.edu; 2 Stony Brook Cancer Center, Stony Brook University Hospital, Stony Brook, NY 11794, USA; mohamed.salama@stonybrookmedicine.edu; 3 Department of Medicine, Stony Brook University, Stony Brook, NY 11794, USA; 4 Department of Biochemistry, Faculty of Veterinary Medicine, Mansoura University, Mansoura 35516, Dakahlia Governorate, Egypt

**Keywords:** non-small cell lung cancer (NSCLC), targeted therapy, chemotherapy, protein kinase C (PKC), drug resistance, epidermal growth factor receptor (EGFR), tyrosine kinase inhibitors (TKI), enzastaurin

## Abstract

Driver-directed therapeutics have revolutionized cancer treatment, presenting similar or better efficacy compared to traditional chemotherapy and substantially improving quality of life. Despite significant advances, targeted therapy is greatly limited by resistance acquisition, which emerges in nearly all patients receiving treatment. As a result, identifying the molecular modulators of resistance is of great interest. Recent work has implicated protein kinase C (PKC) isozymes as mediators of drug resistance in non-small cell lung cancer (NSCLC). Importantly, previous findings on PKC have implicated this family of enzymes in both tumor-promotive and tumor-suppressive biology in various tissues. Here, we review the biological role of PKC isozymes in NSCLC through extensive analysis of cell-line-based studies to better understand the rationale for PKC inhibition. PKC isoforms α, ε, η, ι, ζ upregulation has been reported in lung cancer, and overexpression correlates with worse prognosis in NSCLC patients. Most importantly, PKC isozymes have been established as mediators of resistance to tyrosine kinase inhibitors in NSCLC. Unfortunately, however, PKC-directed therapeutics have yielded unsatisfactory results, likely due to a lack of specific evaluation for PKC. To achieve satisfactory results in clinical trials, predictive biomarkers of PKC activity must be established and screened for prior to patient enrollment. Furthermore, tandem inhibition of PKC and molecular drivers may be a potential therapeutic strategy to prevent the emergence of resistance in NSCLC.

## 1. Introduction

Lung cancer is the most prevalent cancer and the leading cause of cancer-related mortality worldwide, with an estimated 2,093,900 new cases and 1,761,000 deaths annually [1,2]. Due to the initial asymptomatic course of lung cancer, most patients present with locally advanced or metastatic disease at the time of diagnosis. Metastatic lung cancer has significantly limited therapeutic options and is associated with highly unfavorable prognosis. The current clinical outcomes for lung cancer patients are far from satisfactory, and novel treatments must be developed that improve overall survival (OS) [3]. Lung cancer is histologically classified into small cell lung cancer and non-small cell lung cancer (NSCLC). NSCLC accounts for the largest subset of lung cancer cases, roughly 85%, and is further categorized into adenocarcinoma, squamous cell carcinoma, and large-cell carcinoma [4,5].

**Figure 1:**
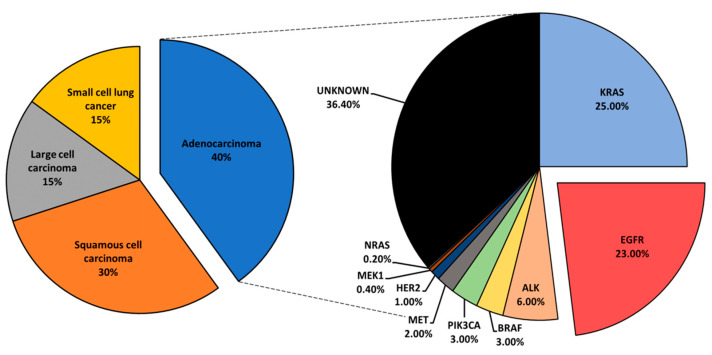
An overview of lung cancer histology and driver mutations in adenocarcinoma patients.

Mutational profiling of lung adenocarcinoma patients reveals Kirsten rat sarcoma viral oncogene (*KRAS),* epidermal growth factor receptor (*EGFR*), and anaplastic lymphoma kinase (*ALK*) as the most prominent oncogenic drivers. Other, less common mutations have been reported and include *BRAF*, *PIK3CA*, *MET*, *HER2*, *MEK1*, and *NRAS* (Figure 1) [6]. Driver mutations define tumor biology and present vulnerabilities that could be exposed via specific inhibition to suppress tumor growth. Driver-directed therapeutics have heralded impressive clinical outcomes, drastically changing the treatment course and the progression-free survival (PFS) of patients who would otherwise be given standard chemotherapy with an estimated median survival of under 12 months [7]. Critically, patients with appropriate biomarkers initially show remarkable responses to targeted therapy. However, nearly all patients relapse with tumors that are no longer sensitive to original treatment, and such an acquired resistance greatly hinders the clinical outcomes of lung cancer patients. Therefore, understanding the mechanisms that drive the emergence of resistance is of interest, and therapeutic approaches that overcome resistance are essential [8].

In addition to the commonly identified driver mutations, other upregulated mediators have been observed in NSCLC. Interestingly, elevated protein kinase C (PKC) isoforms α, ε, η, and ι have been observed in NSCLC and associated with poor prognosis, hinting at a potential role in mediating tumorigenesis [9]. In this review, we will discuss the consequence of PKC regulation in the context of NSCLC, with hopes to elucidate the potential benefits of inhibiting PKCs, likely in tandem with targeted therapy.

## 2. The Protein Kinase C Family

**Figure 2:**
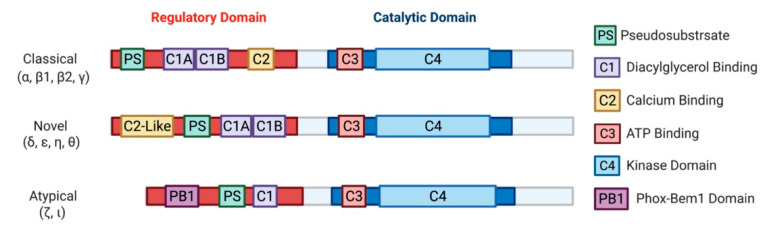
A schematic representation of PKC subfamily structural domains. Distinct PKC isozymes are categorized into classical, novel, or atypical PKCs based on N-terminal regulatory domain structure and have conserved C1-4 domains. Classical PKC α, β1, β2, γ are activated by DAG and calcium through binding with C1A-C1B and C2 domain, respectively. Novel PKC isoforms δ, ε, η, θ are DAG dependent but calcium independent for their activation, as the C2-like domain cannot bind calcium. Atypical PKC ζ, ι do not respond to calcium or DAG. All PKC isozymes have a pseudosubstrate (PS) domain involved in kinase auto-inhibition. The C-terminal catalytic domain is highly homologous between all the PKC isozymes and consists of an ATP binding C3 domain and a C4 kinase domain.

The family of PKC has been extensively reviewed over the years [10,11,12,13,14,15,16,17]. Briefly, PKCs were initially discovered in 1977 by the group of Yasutomi Nishizuka [18]. Later characterization of this novel kinase led to the discovery of three classes: classical (α, β1, β2, γ), novel (δ, ε, η, θ), and atypical (ζ, ι) PKCs [19,20,21,22,23,24,25]. Biochemical analysis of PKCs revealed a highly conserved C-terminal catalytic domain, with a variable N-terminal regulatory domain (Figure 2) [26]. Identification of PKC as a direct effector of diacylglycerol (DAG) defined the primary second messenger function of DAG and connected PKC to the phosphatidylinositol (PI) cycle of signaling [27]. Cytosolic concentrations of second messenger activators of PKC, DAG and calcium, are mediated by phospholipase C, which cleaves phosphatidylinositol 4,5-bisphosphate (PIP_2_) to generates DAG and inositol trisphosphate (IP_3_). IP_3_ further regulates cytosolic calcium levels. The discovery of PKC activation by tumor-promoting agent phorbol 12-myristate 13-acetate (PMA) in 1982 [28] drew attention to this family of kinases based on the plethora of cell biologic responses to PMA and other phorbol esters, warranting extensive research that implicated PKC isozymes in various pathologies, including cancer, heart disease, diabetes, and several neurological diseases [10,11,12,13,14,15,16]. It should be noted that the atypical PKCs, PKCι and PKCζ, are not targets for either DAG or phorbol esters.

**Figure 3:**
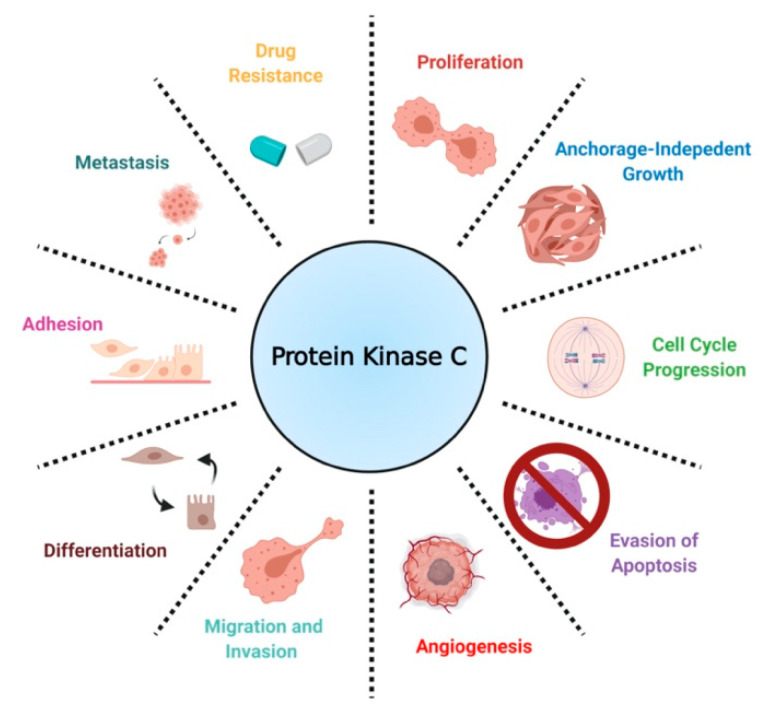
A scheme defining the biological roles of PKC in NSCLC.

In the context of cancer, PKCs are known to regulate several cellular processes, including proliferation, cell cycle progression, angiogenesis, metastasis, apoptosis, and drug resistance (Figure 3) [16]. Contrary to findings implicating PKCs as promoters of cancer progression, a separate body of work has established PKCs as tumor suppressors in various tissues by inducing differentiation and inhibiting anchorage-independent growth, migration, and metastasis. [17,29,30]. Contradictory results on the biological role of PKCs have led to the conclusion that PKC-mediated biology is highly tissue- and isozyme-specific [31].

## 3. Expression, Biological Role, and Prognosis of Protein Kinase C in NSCLC

Bioinformatic analysis using gene expression databases and immunohistochemistry (IHC) analysis of patient tissues have revealed the upregulation of several PKC isoforms in NSCLC compared to normal lung epithelium. 

PKCα is highly expressed in NSCLC. Expression is higher in adenocarcinoma than squamous cell carcinoma [32]. NSCLC cell lines H1355, H157, H1155, H1703, and A549 showed elevated PKCα levels compared to normal human bronchial epithelial cells [33]. Elevated PKCα activity has been observed in A549, PC-9, PC-14, and RERF-LC-MS NSCLC cell lines [34]. Notably, a recent study reported significantly worse OS in lung adenocarcinoma patients expressing relatively high PKCα protein levels [35]. In agreement with this finding, a vast body of work on PKCα has implicated the kinase as a promoter of tumorigenesis in *KRAS* or *EGFR* mutant NSCLC. Antisense oligonucleotide-mediated suppression of PKCα demonstrated antitumor activity in LTEPa-2 and A549 by reducing proliferation and invasive phenotype in tissue culture [36,37]. Furthermore, antisense downregulation of PKCα inhibited tumor growth of A549-inoculated xenografts in vivo [38]. In addition to positively regulating cell proliferation and migration, PKCα is more specifically implicated in cell cycle progression and apoptosis in NSCLC. Antisense downregulation of PKCα and θ in H23 cells resulted in an increased expression of p21, leading to G1 arrest in a p53-independent manner [39]. PKCα has also been implicated in drug sensitivity and resistance acquisition and was reported to mediate doxorubicin sensitivity by phosphorylation of RLIP76 in NSCLC [40,41]. Interestingly, a separate study has linked PKCα to multi-drug resistance gene *MDR1*, suggesting a potential mechanism by which PKC mediates drug sensitivity [42]. The PKC inhibitor, chelerythrine chloride, decreased PKCα mRNA expression and protein levels and sensitized cisplatin-resistant A549 to cisplatin [43]. However, PKC and drug sensitivity findings are not limited to PKCα, but are also reported for other isoforms [44]. Upregulation, increased activity, and tumor-promoting properties of PKCα deem the kinase as a potential marker and therapeutic target in NSCLC patients. Notably, the detection of activated PKCα in serum has been reported to be a potential prognostic marker for lung cancer [45].

PKCδ has been suggested as a therapeutic target for NSCLC. In NSCLC cells with mutant *KRAS*, targeting PKCδ has been shown to inhibit invasion, migration, and colony formation [46]. Moreover, inhibiting PKCδ in NSCLC cells promotes drug-induced apoptosis [33]. In H1299, HSP27 and PKCδ heptapeptide interaction has been linked to drug and radiation resistance [47].

PKCε overexpression has been detected in >90% of NSCLC patient samples via IHC [48]. Low expression of PKCε in healthy tissue has made the isozyme a potential cancer marker [49]. Functionally, PKCε has been linked to enhanced proliferation, cell cycle progression, migration, and evasion of apoptosis in NSCLC. Ectopic expression of dominant-negative kinase-deficient PKCε demonstrated significantly reduced proliferation and impaired anchorage-independent growth in H358, H460, H23, and H157 when compared to vector controls. This was accompanied by G1 arrest as a consequence of enhanced p21 inactivation of cdk2 [48]. Molecular and pharmacological inhibition of PKCε impaired invasiveness of A549 cells in vitro. Knockdown of PKCε by an isoform-specific siRNA downregulated expression and secretion of several metalloproteases, suggesting a possible mechanism for reduced migration. In vivo metastasis models using a stable shRNA-mediated depletion of PKCε in A549 have confirmed in vitro findings. Separately, pharmacological inhibition of PKCε using isoform-specific peptide inhibitor εV1-2 was reported to reduce H358 tumor growth in athymic nude mice [50]. PKCε downregulation impaired the metastatic potential of intravenously inoculated A549 cells [51]. Furthermore, PKCε downregulation has been linked to apoptosis; PKCε- depleted cells expressed elevated levels of several pro-apoptotic genes, such as Bak, and showed a decrease in anti-apoptotic Bcl-2 mRNA expression. Interestingly, a separate study on miR-143, shown to specifically regulate PKCε, also confirmed the tumor-promoting role of PKCε and its implication in apoptosis. miR-143 has been reported to be downregulated in lung cancer, and its suppression enhanced the proliferation and allowed the evasion of apoptosis in A549 and Calu-1 cells [52]. These findings elucidate the potential therapeutic advantage of PKCε inhibition in a subset of lung cancer patients overexpressing the kinase. 

Although little is known about the biological role of PKCη in NSCLC, PKCη protein levels correlated positively with disease stage, and its overexpression has been linked to poor prognosis in lung cancer [53]. A previous study reported an increased risk of death within the first year of diagnosis in NSCLC patients with relatively higher levels of PKCη [54]. Moreover, antisense downregulation of PKCη augmented the antitumor effects of vincristine and paclitaxel in A549 cells [44]. These findings warrant further investigation into the biological consequence of PKCη regulation in NSCLC to expose potential vulnerabilities [55].

PKCι is similarly overexpressed in NSCLC tissues compared to normal lung epithelium [56,57,58,59,60]. Western blot analysis of NSCLC cell lines A549, H520, H1299, H292, ChaGo, and Sk-Mes1 showed high PKCι protein levels [59,61]. Overexpression of PKCι correlated with poor OS of lung adenocarcinoma patients [62]. Moreover, there is a positive relationship between PKCι expression and glucose metabolism. Patients with a higher expression of PKCι and glucose transporter GLUT1 showed a poorer prognosis [63]. Elevated expression of PKCι mRNA and protein levels in NSCLC have been attributed to *PRKCI* gene amplification [57]. Importantly, PKCι has been implicated in NSCLC growth, migration, and anti-apoptotic signaling. Stable expression of kinase-deficient PKCι impaired anchorage-independent growth of A549, H1299, and ChaGo cells [57,61]. In agreement with this finding, a study identifying targets of PKCι revealed that the downregulation of four downstream effector genes, *COPB2*, *ELF3*, *RFC4*, and *PLS1*, suppressed the transformed phenotype of A549 [64]. PKCι-dependent activation of Rac1 resulted in subsequent activation of the PAK/MEK/ERK pathway. Constitutively active Rac1 restored anchorage-independent growth of A549 stably expressing kinase-inactive PKCι [61]. A subsequent study noted Ect2 as another effector, regulated by PKCι. Importantly, the PKCι-regulated phosphorylation of Ect2 was a critical event for the promotion of anchorage-independent growth of H1703 cells [65]. Notably, a study looking at lung adenocarcinoma tissue reported increased PKCι expression in invasive lesions [62]. Pharmacological inhibition of PKCι using atypical PKC inhibitor DNDA increased the apoptosis of H1299 and A549 cells, accompanied by a decrease in pro-survival Bcl-2 and an increase in cleaved caspase-3 [59]. Additionally, PKCι regulates Bcl-x splicing, promoting survival through anti-apoptotic Bcl-x(L) expression [66]. In a recent study, PKCι was shown to phosphorylate ELF3 transcription factor, driving the expression of *NOTCH3*, which, in turn, induced stemness and promoted lung tumor formation in *KRAS*-mutant NSCLC [67]. These findings provide compelling evidence linking PKCι to invasion and metastasis in NSCLC. Therefore, the inhibition of PKCι may be a rational approach to suppressing NSCLC, particularly in specific contexts such as mutant *KRAS-*expressing lung adenocarcinoma [68]. Inhibition of the PKCι-PAK1 pathway significantly reduced cell viability and colony formation of HCC827, H23, and H520 cells, showing efficacy not only in mutant *KRAS* cells, but also mutant *EGFR* (Δ*EGFR)* cell lines [69]. Importantly however, lung adenocarcinoma may develop through both PKCι-dependent and PKCι-independent pathways [70]. Therefore, it is critical to limit PKCι-targeted approaches to PKCι-dependent tumors.

More recently, PKCζ upregulation has also been observed in NSCLC. PKCι and PKCζ are both reported to be downstream effectors of YAP, which regulates the phosphorylation of both atypical PKCs, promoting lung adenocarcinoma tumorigenesis [60]. The specific inhibition of PKCζ was previously shown to regulate NSCLC chemotaxis [71]. Since evidence associates both ι and ζ isoforms with pro-invasive biology in NSCLC, atypical PKC inhibition may be a promising therapeutic approach to impair proregression of lung cancer in patients with elevated or activated PKCι and PKCζ.

Very little is known about the biological roles of PKCβ1, β2, γ, θ in NSCLC. PKCβ promotes angiogenesis in glioblastoma, breast, ovarian, and prostate cancer [72,73,74]. Additionally, PKCβ2 is highly expressed in chronic lymphocytic leukemia and chronic myelogenous leukemia, where the kinase suppresses anti-apoptotic signals [75,76,77,78]. PKCγ expression is mainly limited to neuronal tissues [79]. Furthermore, novel PKC isoform θ is primarily expressed in hematopoietic cells [80,81]. The expression and known biological roles of PKCs in NSCLC are summarized in Table 1.

## 4. Therapeutic Approaches Targeting PKCs in NSCLC

PKCs have been targeted alone or in combination with other agents in clinical trials through the use of potent activators, antisense oligonucleotides, and specific/ non-specific kinase inhibitors.

The most common clinical and pre-clinical approach in regulating PKC activity is by small-molecule inhibition. ATP-competitive PKC inhibitors are mostly non-specific and show off-target effects on alternate PKC isoforms and other closely related serine/threonine kinases. Lack of isozyme-specific inhibitors is a critical limitation associated with this approach. Enzastaurin, a PKCβ inhibitor with in vitro IC50 for PKCα: 39 nM, PKCβ: 6 nM, PKCγ: 83 nM, PKCε: 110 nM, is one of the best-studied inhibitors in NSCLC [85]. Enzastaurin treatment of NSCLC cells H520, Calu1, Calu3, and Calu6 impaired colony-forming capability at clinically attainable concentrations [86]. Two separate phase I clinical trials reported favorable toxicity profiles for enzastaurin [87,88]. Enzastaurin as second- or third-line treatment of NSCLC patients resulted in disease stabilization of a small subset of patients (13%) enrolled in the clinical trial [89]. As enzastaurin was well-tolerated in previous studies, further combination of the inhibitor with cytotoxic agents was recommended. Enzastaurin and pemetrexed synergistically inhibited cell cycle progression, enhanced apoptosis, and modulated signaling by reducing AKT phosphorylation and *VEGF* expression in A549 and SW1573 cells [90]. Enzastaurin, in combination with pemetrexed, was well tolerated in a phase I clinical trials and demonstrated therapeutic efficacy as second-line treatment for patients with advanced NSCLC [91]. Although well-tolerated, enzastaurin and cisplatin-pemetrexed combination as a first-line treatment did not improve PFS or OS of NSCLC patients according to two independent phase II clinical trials [92,93]. In another clinical study assessing PKC inhibitor efficacy in combination with chemotherapy, enzastaurin did not add to the antitumor effects of pemetrexed-carboplatin [94]. A subsequent meta-analysis evaluating additive effects of PKC inhibitors in combination with chemotherapy reported no significant improvement in PFS and OS across all studies and noted additional toxic effects with PKC inhibition [95]. Despite showing pre-clinical success, PKC inhibitors have failed to provide significant benefits in clinical trials. This inefficacy may be explained by the lack of patient stratification based on PKC expression in the clinical trials described above. Most in vitro and in vivo models were conducted on cells with elevated PKC. However, no effort was made to test patients enrolled in clinical trials for PKC levels or predictive biomarkers of PKC activity. Although many factors could explain the discrepancy between pre-clinical and clinical studies, patient treatment without molecular testing is not an optimal approach to assess PKC inhibitor efficacy. It may also be possible that the cytostatic effects of enzastaurin limit the therapeutic potential of chemotherapeutic agents, thus demonstrating little efficacy, as seen in the combination treatment trials described above. 

Tumor-promoting phorbol ester 12-O-tetradecanoyl- phorbol-13-acetate (TPA), bryostatin-1, and bryostatin-2 are potent activators of PKCs. These activators mimic DAG by binding to the C1 domain of classical and novel PKCs [28]. Unlike DAG, however, these activators are not readily metabolized and result in prolonged activation of the isozymes [96]. Bryostatin-mediated activation of PKCs (and less so with TPA) results in the acute degradation of some PKCs following ubiquitination, leading to growth arrest [97,98]. Several studies reported that the treatment of A549 cells with PKC activators results in growth arrest, accompanied by reduced PKC levels and activity [99,100,101,102,103]. TPA was shown to induce G1 arrest in H358 [104] and increased senescence-associated-β-galactosidase marker [105]. Importantly, neither PKC protein nor activity was measured in these studies; therefore, it is not clear if these findings are due to PKC activation or its downregulation following prolonged activation via TPA as previously reported. PKC activators have demonstrated impressive growth suppression of NSCLC cell lines in vitro and have prolonged OS in vivo [106]. Unfortunately, however, bryostatin-1, administered in combination with paclitaxel, showed no significant benefit in patients with NSCLC and showed unfavorable toxicity profiles [107].

Pre-clinical models using antisense oligonucleotide-mediated suppression of PKCα demonstrated significantly impaired NSCLC tumor growth [36,37,38]. In addition, the antisense inhibition of PKCα in subcutaneously injected H460 cells enhanced the antitumor effects of cisplatin [108]. Separately, knockdown of PKCα sensitized cells to several anticancer drugs, including carboplatin and doxorubicin [40,42]. These findings provided a rationale for antisense-directed therapy against PKCα in clinical trials. Initially, a phase I/II study using a PKCα antisense inhibitor LY900003 exhibited antitumor activity when administered in combination with cisplatin and gemcitabine [109]. On the other hand, phase II and phase III clinical trials using a PKCα-specific antisense inhibitor aprinocarsen, in combination with chemotherapy, did not show significant survival benefits and exhibited additional toxic effects [110,111,112]. However, it is worth mentioning that the patients enrolled in these studies were not selected or based on any biomarkers that indicated the prevalence of PKCα overexpression or increased activity. Therefore, the obtained results may not accurately depict therapeutic efficacy as selection of a more appropriate patient cohort is necessary. It has become increasingly clear that, in assessment of targeted therapy, patients must be selected based on host tumor expression of the targeted aberrant gene. 

## 5. PKC-Mediated Resistance Acquisition and Drug Sensitivity in NSCLC

**Figure 4:**
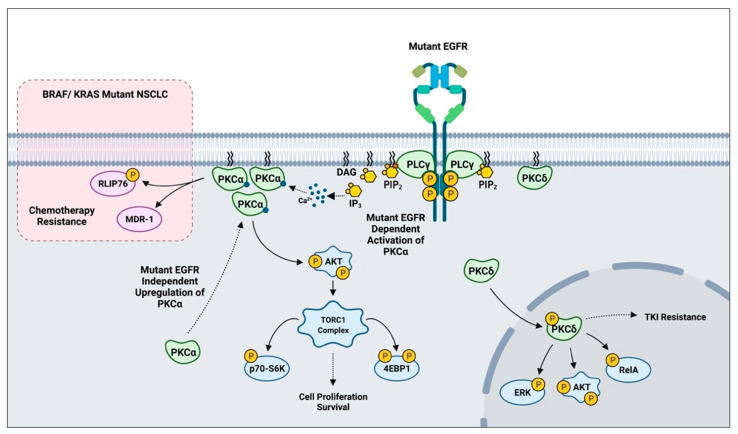
A scheme outlining PKC-mediated drug resistance in *EGFR* and *KRAS* mutant NSCLC.

In addition to the already described biology, PKCs appear to play a role in mediating sensitivity to cytotoxic agents and targeted therapy based on in vitro models. More recent findings suggest that PKCs may also play a crucial role in mediating resistance to tyrosine kinase inhibitors (TKI). One study reported that erlotinib-resistant H1650-M3 cells expressed significantly higher PKCα and had lower PKCδ mRNA levels relative to the parental H1650. RNAi and small molecule inhibition of PKCα sensitized H1650-M3 to erlotinib, a TKI inhibitor of Δ*EGFR*. Importantly, however, a viral-mediated stable overexpression of PKCα did not affect H1650 sensitivity to erlotinib. This strongly suggests that PKCα alone is not sufficient to induce erlotinib resistance. On the other hand, viral-mediated overexpression of PKCδ moderately increased H1650-M3 sensitivity to erlotinib [82]. A subsequent study combining PKC inhibitor chelerythrine chloride and erlotinib in the treatment of NSCLC A549 and SK-MES1 cells reported a significant synergy in impairing cell viability, colony formation, tumor growth in xenografts, and enhanced apoptosis. Importantly, however, the concentrations of erlotinib used in the experiments (2.5–20 μM) typically exceeded the therapeutic range (1–2 μM), and the cell lines used in the study have wild-type *EGFR* [83]. A more recent study evaluating *EGFR*-TKI resistance reported PKCδ to be necessary and sufficient to induce resistance, and downregulation of the kinase via molecular and pharmacological approaches sensitized TKI-resistant cells to erlotinib. Although contradictory to the previous study in H1650-M3, an shRNA-mediated downregulation of PKCδ sensitized H1650 and TKI-resistant HCC827-GR cells to gefitinib, another *EGFR* TKI. Importantly, ectopic expression of PKCδ was sufficient to induce resistance in TKI-sensitive HCC827 and H3255. Mutations in the nuclear localization signal that sequestered PKCδ in the cytoplasm impaired PKCδ- induced gefitinib resistance and attenuated phosphorylation of ERK, AKT, and RelA in isolated nuclear fractions. Clinically, PKCδ upregulation correlated negatively with OS in TKI-treated Δ*EGFR* NSCLC patients [84]. Interestingly, our recently published work has demonstrated that there is a Δ*EGFR*-independent selection for high PKCα protein expression in NSCLC, and a Δ*EGFR*-dependent activation of PKCα that translates to constitutive downstream signaling to AKT/mTOR pathway [113]. In *BRAF*/ *KRAS* mutant NSCLC, PKCα was shown to modulate sensitivity to chemotherapy via mediating *MDR1* expression and RLIP76 phosphorylation (Figure 4) [40,41,42]. These findings suggest that PKC α and δ inhibition, in the context of mutant driver genes such as *EGFR* and *KRAS*, may be a novel approach to address TKI-sensitivity and resistance to chemotherapy in NSCLC.

## 6. Conclusions

PKC mRNA and protein levels are shown by several reports to be upregulated in NSCLC, and specific isozymes have been implicated as mediators of drug sensitivity in NSCLC. The implication of these isozymes in tumor-promoting biology and drug resistance strongly suggests that PKC inhibition may be an effective therapeutic approach for NSCLC. Despite showing impressive in vitro and in vivo antitumor effects, PKC inhibitors failed to significantly improve clinical outcomes in NSCLC [114]. This could be partly attributed to the lack of appropriate biomarkers for PKC inhibitor efficacy. Therefore, to effectively assess PKC inhibitor efficacy in NSCLC, future clinical trials should be focused on patients with upregulated PKC levels and the relevant biomarkers of increased PKC activity. More importantly, recent research has shown that specific PKC isozymes may play a role in mediating resistance acquisition to TKI and chemotherapeutic agents in NSCLC. Although approaches targeting PKCs alone in NSCLC have yielded unsatisfactory results, it may be plausible that the inhibition of PKCs, in conjugation with other driver-targeted treatment, may present a potential benefit in overcoming resistance acquisition to cancer therapy.

## Figures and Tables

**Table 1:** Proposed biological roles of distinct PKC isozymes in NSCLC.

Isozyme	Biological Roles	References
PKC α	Promotes proliferation, invasion, migration, cell cycle progression, evasion of apoptosis, drug resistance	[36,37,38,39,40,41,42,82,83]
PKC β_1,_ β_2_	Unknown	
PKC γ	Not expressed	
PKC δ	Mediates drug sensitivity, invasion, cell survival	[33,46,47,82,84]
PKC ε	Promotes proliferation, invasion, migration, cell cycle progression, anchorage-independent growth, evasion of apoptosis	[48,50,51,52]
PKC η	Mediates drug sensitivity	[44]
PKC θ	Not expressed	
PKC ζ	Chemotaxis	[71]
PKC ι	Promotes proliferation, invasion, migration, anchorage-independent growth, evasion of apoptosis, stemness, glucose metabolism	[57,61,62,63,64,65,66,67,68,69]
